# Late Open Conversion with Graft Replacement after Endovascular Aortic Repair along with Removal of a Giant Ovarian Tumor: A Case Report

**DOI:** 10.70352/scrj.cr.25-0014

**Published:** 2025-08-26

**Authors:** Takuma Mikami, Daisuke Akashi, Chikara Shiiku

**Affiliations:** 1 Department of Cardiovascular Surgery, National Hospital Organization Obihiro Hospital, Obihiro, Hokkaido, Japan; 2 Department of Gynecology, Obihiro-Kosei General Hospital, Obihiro, Hokkaido, Japan

**Keywords:** abdominal aortic aneurysm, endovascular aortic repair, endoleaks, late open conversion, giant ovarian tumor, octogenarian

## Abstract

**INTRODUCTION:**

There are many reports of late open conversion after endovascular aortic repair (EVAR). Herein, we report the case of an octogenarian patient with a giant ovarian tumor who underwent ovarian tumor resection and open conversion with graft replacement simultaneously via laparotomy.

**CASE PRESENTATION:**

An 86-year-old woman underwent EVAR 7 years ago. After surgery, persistent type II endoleaks from the lumbar arteries were detected. The diameter of the aneurysmal sac gradually increased. Although we attempted to perform coil embolization of the lumbar arteries one year ago, the procedure was unsuccessful, and type II endoleaks persisted, further dilating the aneurysmal sac. One month earlier, the patient experienced lower abdominal pain, which was thought to be a symptom of an ovarian tumor. Surgery to remove the ovarian tumor was considered. Although open conversion was considered, laparotomy was difficult due to the presence of the giant ovarian tumor. Therefore, we decided to perform open conversion with aortic graft replacement in addition to salpingo-oophorectomy. Surgery was performed via a median laparotomy, and graft replacement of the infrarenal abdominal aortic aneurysm was performed after salpingo-oophorectomy. The postoperative course of the patient was uneventful, and she was discharged.

**CONCLUSIONS:**

We performed a salpingo-oophorectomy and late open conversion with graft replacement after EVAR in an octogenarian patient with a giant ovarian tumor and achieved favorable results.

## Abbreviations

AAA
: abdominal aortic aneurysm
EVAR
: endovascular aortic repair
T2EL
: type II endoleak

## INTRODUCTION

Favorable perioperative results have been reported for endovascular aortic repair (EVAR).^1,2)^ However, postoperative endoleaks may lead to enlargement of the aneurysmal sac and require reintervention. A persistent type II endoleak (T2EL), caused by the backflow from branches of the aneurysm, has been reported as a risk factor for enlargement of the aneurysmal sac.^3,4)^ If endovascular therapy is not possible as a reintervention, open conversion is indicated. However, in octogenarians, open conversion is often impossible because of frailty and comorbidities. In addition, open conversion in patients with giant ovarian tumors is not usually performed at the same time as surgery because there is a risk of infection of the graft if the ovarian tumor ruptures during surgery. Herein, we report the case of an octogenarian with a giant ovarian tumor who underwent salpingo-oophorectomy and open conversion with graft replacement after EVAR simultaneously.

## CASE PRESENTATION

**Fig. 1:**
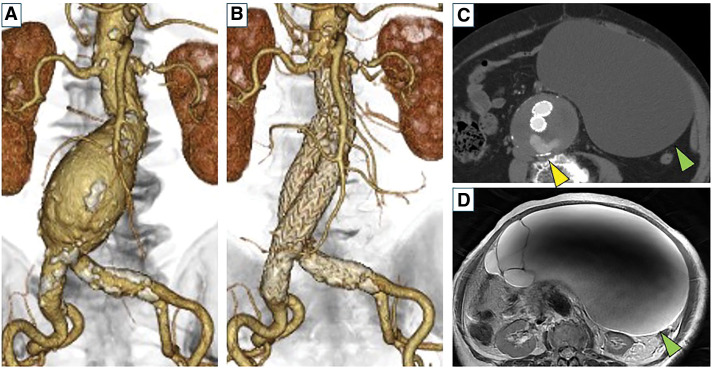
CT radiographs acquired prior to surgery. (**A**) Contrast-enhanced CT radiograph acquired before EVAR was conducted 7 years ago. A fusiform-shaped abdominal aortic aneurysm was visible below the renal arteries. The inferior mesenteric artery was occluded before EVAR. (**B**) Contrast-enhanced CT radiograph acquired one week after EVAR. EVAR was performed using Excluder C3 (W. L. Gore & Associates, Flagstaff, AZ, USA), in accordance with the manufacturer’s instructions for its use. The preoperative diameter of the abdominal aortic aneurysm was 50 × 50 mm. (**C**) Axial image acquired via contrast-enhanced CT one year ago and before coil embolization, showing type II endoleak from the lumbar artery (yellow arrowhead). The abdominal aortic aneurysmal sac had expanded to 62 × 74 mm. A giant ovarian tumor was found in the abdominal cavity (green arrowhead). The volume of the ovarian tumor at the time of imaging was 2395 cm^3^. (**D**) T2-weighted radiographs acquired via abdominal MRI showing a hyperintense cystic lesion measuring 25 × 21 × 15 cm in the abdominal cavity. There was no internal solid component or wall thickening (green arrowhead), and no obvious malignant findings were observed. EVAR, endovascular aortic repair

**Fig. 2:**
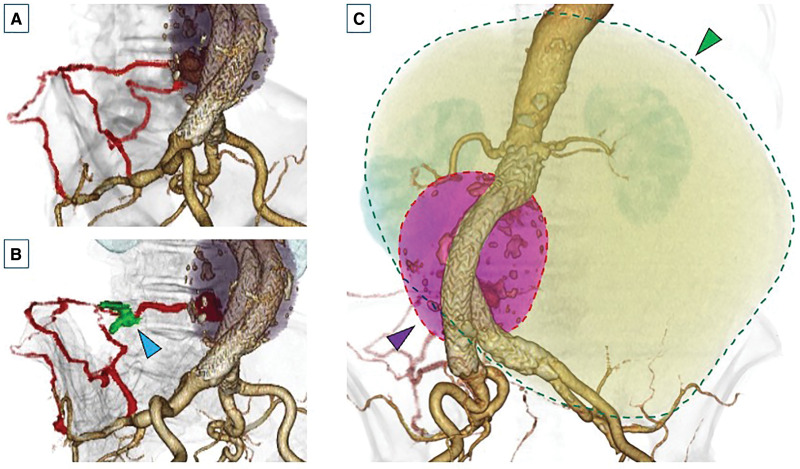
CT radiographs acquired one year ago. (**A**) Contrast-enhanced CT radiograph acquired one year ago before coil embolization, showing that the right L4 lumbar artery, which was the cause of the type II endoleaks, had developed collateral circulation from the right superior gluteal artery. (**B**) Contrast-enhanced CT radiograph acquired after coil embolization, showing that the coil advanced only halfway through the catheter and was embolized in the middle of the path (blue arrowhead). (**C**) Preoperative contrast-enhanced CT radiograph showing the diameter of the aneurysm sac to be 64 × 81 mm (red dashed line, purple arrowhead). The giant ovarian tumor had expanded, and its volume was 5381 cm^3^ (green dashed line, green arrowhead). The abdominal aortic aneurysm was displaced to the right side by the giant ovarian tumor.

An 86-year-old woman underwent EVAR for an infrarenal abdominal aortic aneurysm (AAA) 7 years prior (**Fig. 1A** and **1B**). The diameter of the aortic aneurysm before the initial EVAR was 50 × 50 mm. Six lumbar arteries branched from the AAA, 2 each at L2, L3, and L4. The diameter of the L4 lumbar artery was 2.1 mm. The inferior mesenteric artery was also patent, with a diameter of 2.3 mm. Branch embolization was not performed before or during the initial EVAR. The preoperative proximal neck length was 25 mm. Her comorbidities included hypertension, dyslipidemia, chronic kidney disease, and an ovarian tumor. Her clinical frailty scale score was 4. After EVAR, a persistent T2EL was observed in the right L4 lumbar artery, and the aneurysmal sac gradually expanded to 62 × 74 mm (**Fig. 1C**). The ovarian tumor also gradually expanded, and MRI results indicated a mucinous cystadenoma (**Fig. 1D**). Although we attempted coil embolization of the right L4 lumbar artery via the right superior gluteal artery one year ago, the catheter could not reach the origin of the right L4 lumbar artery. Therefore, coil embolization was performed along the route (**Fig. 2A** and **2B**). However, the T2EL persisted, and the aneurysmal sac continued to expand slowly because the procedure was unsuccessful. The results of the preoperative laboratory tests were as follows: white blood cell count 3700/μL, hemoglobin 9.5 g/dL, creatinine 1.15 mg/dL, estimated glomerular filtration rate 33.3 mL/min/1.73 m^2^, C-reactive protein 0.36 mg/dL, cancer antigen 125 (CA-125) 93 U/mL (reference value 0–35), CA19-9 5.0 U/mL (reference value 0–37), and carcinoembryonic antigen 3.4 ng/mL (reference value 0–5). An ovarian tumor was detected with the absence of any findings suggestive of malignancy at the gynecology department of another hospital. However, surgery was ruled out owing to the advanced age of the patient. Although we considered open conversion to treat the aneurysmal sac enlargement after EVAR, it was difficult to perform open conversion via a median laparotomy, including the retroperitoneal approach, because of the giant ovarian tumor. Therefore, we conducted follow-up observations for the aneurysmal sac enlargement after EVAR. One month earlier, the patient experienced lower abdominal pain, and CT showed that the AAA was displaced to the right by the giant ovarian tumor (**Fig. 2C**). In addition, the aortic aneurysm had expanded to 64 × 81 mm in diameter, and the length of the proximal neck decreased to 15 mm because of the expansion of the aneurysmal sac. The ovarian tumor had grown from 15 to 25 cm in a year, and because there was a possibility that the giant ovarian tumor was causing the lower abdominal pain, a gynecologist considered a salpingo-oophorectomy. Therefore, we decided to perform salpingo-oophorectomy and open conversion with graft replacement simultaneously.

**Fig. 3:**
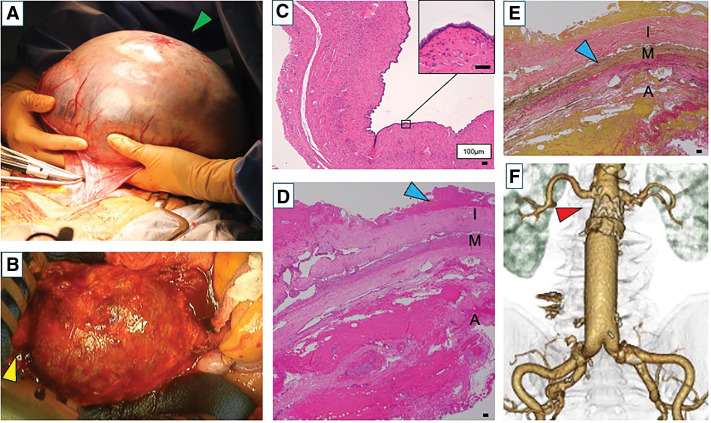
Intraoperative images, histochemical micrographs, and CT radiographs of the patient during and after surgery. (**A**) The intraoperative image shows the ovarian tumor being removed from the abdominal cavity (green arrowhead). (**B**) Intraoperative image acquired at the time when the abdominal aortic aneurysm was exposed by incising the retroperitoneum. The aortic aneurysmal sac was expanded, causing shortening of the proximal neck (yellow arrowhead). (**C**) Representative hematoxylin–eosin staining micrograph showing a monolayer of mucinous epithelium lining the inner surface of the cyst. The findings were consistent with mucinous cystadenoma. No atypical epithelium was observed or findings suggestive of malignancy were observed (magnification: ×40; square: ×200). (**D**) Hematoxylin–eosin staining micrograph of the wall of the resected abdominal aortic aneurysm. No inflammatory cell infiltration was observed, but hyaline degeneration and calcification of the intima were observed (blue arrowhead). These findings were consistent with an atherosclerotic aortic aneurysm. (**E**) Elastica van Gieson staining of the resected abdominal aortic aneurysm showed severe atrophy of the media elastic fibers was observed (blue arrowhead). The findings were consistent with atherosclerotic aortic aneurysm. (**F**) Contrast-enhanced CT one week after open conversion with graft replacement showed that the proximal stent graft was partially preserved (red arrowhead) and replaced with the new graft. A, adventitia; I, intima; M, media

Surgery was performed via a median laparotomy. No adhesions were observed in the abdominal cavity. Although the tumor originated from the right ovary and had undergone torsion once, blood flow was maintained. After detorsion, a right salpingo-oophorectomy was performed (**Fig. 3A**). Subsequently, the retroperitoneum was incised to expose the AAA (**Fig. 3B**). Preoperative CT results revealed that the aneurysmal sac was enlarged due to T2EL. Hence, the aortic aneurysmal sac was opened without cross-clamping the aorta. After removing the thrombus within the aneurysmal sac, a strong backflow was observed from the right L4 lumbar artery, in line with the CT findings. Although oozing from the aneurysmal wall itself was also strong, no other endoleaks were observed. The abdominal aorta with the stent graft was clamped below the bilateral renal arteries, and the bilateral internal and external iliac arteries were clamped. The graft we selected was a 20 × 10 mm J-Graft Bifurcated (Japan Lifeline, Tokyo, Japan). The proximal stent graft was preserved and anastomosed with the new graft using a felt strip placed on the outside. The distal stent graft legs were completely removed and anastomosed with the new graft legs at both common iliac arteries. The operative time was 175 min, and the intraoperative blood loss was 3264 mL. The giant ovarian tumor we removed weighed 5160 g. Cytology of the ascites fluid collected during the surgery and histopathologic examination of the giant ovarian tumor revealed no malignant findings (**Fig. 3C**). Histopathologic findings of the aneurysmal sac showed no evidence of either an inflammatory or infected aortic aneurysm (**Fig. 3D** and **3E**). There were no postoperative complications. Postoperative CT scan results showed that the AAA was completely replaced (**Fig. 3F**). The patient was discharged from the hospital 10 days after the surgery.

## DISCUSSION

Here, we report a case in which open conversion was performed for aneurysmal sac enlargement due to a T2EL after EVAR in a patient with a large ovarian tumor. Since the aneurysm sac had expanded after the failure of endovascular therapy for T2EL, open conversion was desirable; however, this was difficult because of the giant ovarian tumor. In this case, the patient experienced abdominal pain preoperatively, which was thought to be due to the ovarian tumor. Therefore, it would have been preferable to perform an open conversion after salpingo-oophorectomy. However, it would have been difficult to perform 2 laparotomies because the patient was elderly. Therefore, we decided to perform the surgeries simultaneously. The most important aspect of this surgery was the removal of the ovarian tumor without rupturing it, as this could contaminate the abdominal cavity. The ovarian tumor causing the abdominal pain may have adhered due to inflammation, so we asked a gynecologist to safely remove the tumor and perform surgery for the AAA.

EVAR has been reported to have favorable perioperative results; however, its long-term results are poor compared to open aneurysm repair.^5–7)^ One reason for this outcome is postoperative aneurysmal sac enlargement and rupture due to endoleaks. Branch arteries may be embolized prior to EVAR to prevent T2ELs. However, when the initial EVAR was performed in this case, there was insufficient evidence that T2EL was a risk factor for reintervention. Therefore, branch embolization was not performed. Endovascular therapy is often selected as the first reintervention for endoleaks after EVAR. In this patient, collateral circulation was detected in the right L4 lumbar artery via the right superior gluteal artery, and it was determined that coil embolization by endovascular therapy was possible. Hence, reintervention was performed one year ago. However, the targeted root of the right L4 lumbar artery could not be embolized, and T2EL persisted, further enlarging the aneurysmal sac even after endovascular therapy. It has been reported that the results of endovascular therapy for T2EL are poor and residual T2EL remains in approximately 50% of cases, requiring further re-intervention.^3)^ If reintervention via endovascular therapy is not feasible, open conversion is indicated. In the patient described here, coil embolization was attempted on the lumbar artery, but the procedure was incomplete. After coil embolization, T2EL persisted, and the aneurysmal sac enlarged. Therefore, open conversion was desirable as the next reintervention. However, open conversion was challenging via either the median or the retroperitoneal approach due to the presence of the giant ovarian tumor. Although the ovarian tumor was expanding, MRI results showed no obvious malignant findings. In addition, there was no torsion or other organ displacement, and the gynecologist did not advise surgery because the patient was old and frail. Therefore, since the patient did not wish to undergo surgery, the aneurysmal sac enlargement after EVAR was also observed during follow-up. Afterward, she was found to have lower abdominal pain caused by the giant ovarian tumor. A gynecologist considered surgery to remove the giant ovarian tumor. Laparoscopic surgery is commonly used to treat benign ovarian tumors. In this case, it would have been possible to use a laparoscopic technique to aspirate the tumor contents and then remove the tumor. We also considered adding lumbar artery ligation to ovarian tumor resection via laparotomy. However, poor outcomes have been reported following branch ligation alone.^8)^ Therefore, in this case, open conversion was preferable as an additional reintervention for the enlargement of the aneurysmal sac after EVAR. Hence, we decided to perform a median laparotomy, which allows for addressing both the ovarian tumor and aneurysmal sac enlargement after EVAR at the same time.

Late open conversion after EVAR includes preserving the stent graft as well as graft replacement. Compared to late open conversion with graft replacement, favorable results have been reported for late open conversion with endograft preservation for T2EL after EVAR, in which the culprit branch arteries leading into the aneurysmal sac are ligated.^9–11)^ In this case, laparoscopic salpingo-oophorectomy and branch ligation were also considered. Although this technique avoids systemic heparinization and may reduce intraoperative blood loss, there are reports of aneurysmal sac re-enlargement after surgery.^11)^ Conversely, we have reported that open conversion with graft replacement after EVAR had excellent results when it was an elective surgery.^11)^ In addition, it would be difficult to perform a re-laparotomy considering the patient’s age and frailty if re-enlargement of the aneurysmal sac occurs after open conversion with graft preservation. Therefore, despite the advanced age and frailty of the patient, we chose open conversion with graft replacement as there was little possibility of further additional reintervention. The results were robust. Since the giant ovarian tumor was accompanied by abdominal pain, the possibility of cervical torsion of the tumor or surrounding adhesions could not be ruled out. In addition, whether an ovarian tumor is benign or malignant is determined by postoperative histopathologic diagnosis. If the tumor is malignant and ruptures during surgery, additional surgery such as a total hysterectomy or additional chemotherapy may be required, and there is also a possibility of recurrence of the ovarian tumor. Therefore, to avoid reoperation and chemotherapy, it was important to remove the ovarian tumor without rupturing it. Hence, we decided to perform the surgery with the cooperation of a gynecologist. The postoperative course was uneventful, and the removal of the giant ovarian tumor proved to be a good intervention that improved the activities of daily living of the patient by increasing her food intake.

## CONCLUSIONS

We were able to achieve favorable results in the case of an octogenarian with a giant ovarian tumor who had an enlarged aneurysmal sac by carrying out salpingo-oophorectomy via median laparotomy and open conversion with graft replacement for T2EL after EVAR.

## DECLARATIONS

### Availability of data and materials

The dataset supporting the conclusion of this article is included within the article.
